# Lime Phosphates

**Published:** 1874-03

**Authors:** J. M. Porter


### LIME PHOSPHATES.

### DOES THE ADMINISTRATION OF THE LIME PHOSPHATES AID IN THE DEVELOPMENT OF GOOD TEETH?

BY J. M. PORTER, D. D. S.

Read before the Ohio State Dental Society at the Eighth Annual Meeting, December, 1873.

This subject having been selected by the Executive Committee for discussion, and being a question upon which members of the dental profession have entertained a variety of of opinions, I do not expect to offer any new theories, but to point out some of the common errors in the treatment generally adopted in the administration of the phosphates. I do not believe that the administration of the lime phosphates, without preliminary treatment, will have any perceptible effect. I am fully aware that this proposition is generally regarded as erroneous.

It is universally true of patients suffering for a want of lime salts that they do not suffer because there is not a sufficient amount of lime salts in the food, but because of a want of digestive and assimilative power. This being true, it is necessary to institute a course of tonic treatment, as the diseased conditions clearly indicate this to be the proper course, and because the lime salts are not readily assimilated. If no other reasons could be given, those already suggested would clearly indicate that to expect the appropriation of the lime phosphates without paving the way for their reception would end in disappointment.

Now, the question is, what shall be the course of treatment pursued? The only correct way to answer this question is to diagnose your case and learn why your patient needs treatment. In examining this class of patients, you will generally discover that your patient needs treatment to increase nervous tone, and to rouse up the circulation.

In such case you will find the following a valuable prescription:

| | |
|---|---|
| ℞ Arsenic, | |
| Strychnia, | aa grs. |
| Pyro. Phos. of Iron | iij " |
| Phos. Acid | i fl. ℥ |
| Simple Syrup | ii " |

Take a teaspoonful in an equal amount of water after each meal.

In some cases you will find the preliminary treatment sufficient to produce such systemic conditions, as will insure the appropriation of the lime phosphates, but in many cases something more is necessary. For, if we expect an element which is not readily assimilated to have a desirable effect, we must have the secretory and excretory functions perform their full duty. To accomplish this result a hydragogue cathartic is indicated. For this purpose the following prescription may be used:

| | |
|---|---|
| ℞ Jalap, | |
| Calomel, | aa, v grs. |
| Elatirium, | i " |

After giving a hydragogue cathartic, then, and generally not till then, will the patient be in such condition as to justify you in expecting the desired results from the administration of the lime phosphates. After bringing about these results, your own judgment must guide you in the treatment next indicated.

Our first proposition was that preliminary treatment had to be instituted before any beneficial effects would result from administering the lime phosphates. We have cited two reasons for the support of the above proposition. The first one was the fact that a surplus amount of lime salts are being thrown off daily by the kidneys, and in the saliva and other secretions; proving conclusively that the trouble is not caused by a deficient amount of lime phosphates in the food, and clearly pointing to imperfect assimilation as the cause of trouble.

The other reason given for preliminary treatment was the conceded fact that the lime phosphates are not readily assimilated.

The same feature is not only true of elements that are not readily assimilated, but is also true of elements that are readily assimilated. For example, take iron. We all know that iron is very readily appropriated and that its effects are very readily perceptible, and notwithstanding this fact, there are conditions presented where iron will not have any perceptible effect, even after it had been prescribed for weeks in succession. For instance, take chlorosis, there are conditions presented that universally prevent any beneficial effects resulting from the administration of iron. I have seen cases of chlorosis combined with general ænemia where no effect could be produced by giving iron, until after preliminary treatment; consisting of strychnia, quinine and other tonic treatment.

Now when this is true of an element that is readily appropriated, it most certainly is true of an element, or of elements that are not readily assimilated. We have always opposed the administration of specific elements, and always oppose treating specific conditions, as many do. While at the same time they ignore the cause or causes producing the abnormal condition they are endeavoring to relieve. When we attempt to crowd nature, we should bear in mind that the animal economy will appropriăte no more than the vital principal stimulating the organism will permit, and so long as our patients differ in temperament, so long will we find the same variety in the physical characteristics, and pathological condition of their dental organs.

Remember that even good digestion does not prove perfect assimilation and oxidation, and that a multitude of conditions may exist to prevent the elements necessary to sustain healthy conditions, from ever reaching the inner chambers where they are most needed, and where they are least liable ever to go. This is especially true of the bones and teeth. In support of this assertion we will simply remind you that a reversal of the forces that carried nourishment in, may carry it out again, unoxidized, and without its having ever accomplished the good results we had anticipated.

We do not object to the administering of the lime phosphates, but when you pretend to get the best results from giving a specific element, without paying any attention to the conditions that brought about the necessity of using the element, then we will be forced to conclude that cause is not to be taken into account in the treatment of disease, a conclusion we are not yet ready to subscribe to. The study of the many causes tending to produce these defects, and the treatment indicated in each case is exceedingly interesting, and if the dental profession expect to be recognized as being more than mere mechanics; a much larger number will have to take a higher position than their present inclinations would seem to indicate. This is especially the duty of the young men, as in a few years they will be expected to diagnose and treat many abnormal conditions which the mass of the profession do not at this time attempt to control.

The time has come for the mass of the profession to know more of general systematic conditions, both in health and disease, and until they do possess this knowledge, they should not complain of not being recognized, and counseled by physicians. Merit in any direction will always be recognized in a substantial manner, and earnest effort never results in failure.

